# Responses of α-amylase and protease activity to chemical agents and metallic salts in barley seeds (*Hordeum vulgare* L.)

**DOI:** 10.1016/j.heliyon.2025.e42056

**Published:** 2025-01-16

**Authors:** Amin Hossain, Umma Fatema Shahjadee, Abu Tareq Mohammad Abdullah, Mohammad Nazrul Islam Bhuiyan, Anjum Zerin Rupa

**Affiliations:** a Institute of Food Science and Technology (IFST), Bangladesh Council of Scientific and Industrial Research (BCSIR), Dhaka, 1205, Bangladesh; b Dhaka Laboratories, Bangladesh Council of Scientific and Industrial Research (BCSIR), Dhaka, 1205, Bangladesh

**Keywords:** Barley, Chemical agents, Metallic salts, Enzyme activity

## Abstract

In modern agriculture, the enzymes inhibition by chemical agents and environmental pollutants accounts for a significant threat to crop health and productivity. Enzymes play a crucial role in maintaining homeostasis in the metabolic processes that sustain life. Understanding what regulates enzyme activity is crucial for many scientific and industrial endeavors. The purpose of this research work was to examine how different chemical agents, and metallic salts affected the activity of two important food enzymes like α-amylase and protease in barley. These studies compared the effects of several chemical treatments applied to barley seeds, including urea, ethylenediaminetetraacetic acid (EDTA), acetic acid, and a wide range of metallic salts. To determine the impact of each chemical on the stability of α-amylase and protease enzyme activity using standard assay procedures. The activities of α-amylase and protease were inhibited by increasing urea concentration, eventually eliminating them at 8 M urea. The enzymes lost their activities completely at 0.50 M EDTA. Treatment with higher acetic acid concentrations decreased their activities, but they retained 20.46 ± 1.06 % and 17.38 ± 1.09 % after treating with 20 % acetic acid. The application of CaCl_2_ led to a progressive increase for both the enzyme activities, but the maximum increases were observed 137.26 ± 1.42 % and 135.65 ± 1.17 % due to 0.50 M Ca^2+^. In the presence of Mn^2+^ and Mg^2+^ salts, enzyme activity increased notably. In contrast to K^+^ and Na^+^, which have negligible or no inhibitory effects but Zn^2+^, Cu^2+^, and Fe^2+^ considerably reduce the activity of both enzymes. According to the findings, the present research could be created with the scope of potentially identifying ways to maintain their activity for agricultural, industrial and also scientific applications.

## Highlights

• The study examines how various chemicals and metallic salts affect α-amylase and protease activity in barley seeds.
• Interestingly, CaCl_2_ increases enzyme activity gradually while urea, EDTA and acetic acid decrease it.
• Metallic salts like Mn and Mg enhance enzyme activity; Zn, Cu, and Fe inhibit it, while Na and K have minimal or no effects.
• These results show the complex function of chemicals, metallic salts, and enzymes, aiding biotechnology and agriculture.

## Graphical abstract

**Figure:**
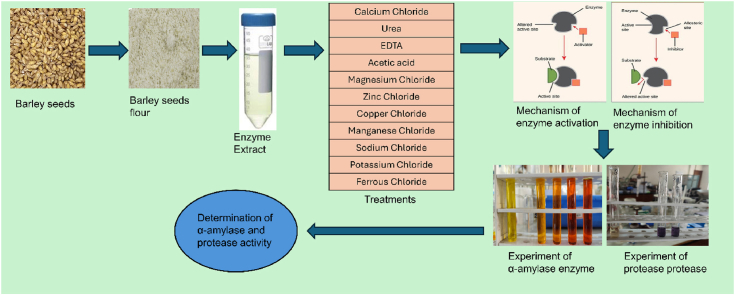

## 1 Introduction

Barley, also known as *Hordeum vulgare* L., is a significant global grain crop, the fifth most common cereal crop. Cultivated during winter, it serves multiple purposes, including fodder utilization and soil fertility enhancement [1,2]. Barley, a cereal crop, has evolved from being primarily used for human consumption to include animal feed, malt products, and human food. Over 60 % of barley is cultivated for animal feed production [3]. It contains various nutritional components, including starch, protein, free lipids, β-glucans, and minerals. Its starch content ranges from 65 % to 68 %, protein from 10 % to 17 %, free lipids from 2 % to 3 %, and β-glucans from 4 % to 9 %. Barley also contains minerals and dietary fiber, with a portion classified as soluble dietary fiber [4,5].

Enzymes are vital constituents of animals, plants, and microbes, including fungus, bacteria, and yeast. Microbial enzymes have been the subject of extensive research; however, until now, research has also focused on enzymes that are present in plant seeds and beans, such as barley, wheat, sorghum, maize, mug beans, and soybeans [[6], [7], [8]]. Some seeds, like barley, store the most crucial enzymes, like amylases and proteases, in their aleurone layer. Generally dormant in nature, seeds activate these enzymes through the imbibition process in contact with water, and the aleurone layer also secretes and produces new enzymes [9,10]. The bulk of α-amylase enzymes, which break down starch, are located in barley seeds [11]. On the other hand, the three most common types of proteases found in plants are cysteine (CPs), serine (SPs), and aspartic (APs). In the barley seeds, cysteine proteases which are cathepsin L-like (EP-A and EP-B), cathepsin H-like have mainly been identified. Besides these, other papain-like cysteine proteases are also found in barely seeds [8,10]. These two hydrolytic enzymes are crucial for seed germination, which is needed for the regeneration of flowering plants. These enzymes are also essential for industrial grain malting [12]. The molecules of barley starch are converted into simple sugars by α-amylase whereas proteins are broken down into small peptides and amino acids by protease. Barley seeds use these as nutrients for the development of their embryo [13]. The α-amylase and protease enzymes are widely used for commercial purposes in the world. The hydrolysis of starch by amylases drives several industrial operations, including the production of glucose syrups, bread, and brewing whereas proteases are used in food, leather, detergent, and pharmaceutical industries [14,15].

Gluten, a protein in wheat, barley, and rye, causes Celiac disease, an autoimmune illness. α-amylase and protease can improve gain composition in cereal like wheat, barley, rye and related hybrids [16]. Amylase and protease are crucial enzymes in cellular metabolism, catalyzing the degradation of macromolecules into small building blocks for energy production or cell biomolecule synthesis [17]. Biologically important enzymes like amylase and protease can have their activity regulated by ions like manganese, magnesium, and calcium. These ions can stabilize enzyme structures, improve substrate binding, or function as cofactors to enhance the activity of enzymes. In contrast, inhibitory effects can be seen with chemical agents such as urea, which can denature proteins, or EDTA which may bind metal ions essential for enzymatic activity.

The research places particular emphasis on two crucial enzymes, namely α-amylase and protease, within the specific framework of barley (*Hordeum vulgare* L.). Investigating plant enzymes is necessary for comprehending their functioning and potential uses. Researchers employ many methodologies to extract, purify, and characterize plant enzymes, enabling a more profound understanding of their characteristics and mechanisms [18,19]. Scientists are discovering methods to modify enzymes to improve plant growth and agricultural output while also utilizing plant enzymes in industrial applications such as biofuel and textile manufacture [20,21]. So, it is essential for exploring the impact of chemical agents and metallic salts on the activity of α-amylase and protease enzymes. This study looked at how chemical agents and metallic ions affect the function of enzymes in barley, specifically α-amylase and protease enzymes. The research also aimed to understand the effects of different chemical treatments on these enzymes, enhancing our understanding of the complex relationships between chemical agents, metallic ions, and enzymatic activities.

## 2 Materials and methods

### 2.1 Sample collection

BARI Barley-7 was purchased from Bangladesh Agricultural Research Institute (BARI). It exhibited great production (about 3 tons per hectare) and another notable characteristic was its absence of husk. In addition, The cultivation of barley-7 variety is increasing in Bangladesh day by day.

### 2.2 Materials and reagents

Analytical grade for each reagent was used for this study. Maltose (Merck, Germany) and leucine (Sigma-Aldrich, USA) were used as standards. Haemoglobin (Sigma-Aldrich, USA) and starch (Merck, Germany) were utilized as the substrates for enzyme. Disodium hydrogen phosphate dihydrate, potassium dihydrogen phosphate (Merck, Germany), and citric acid (Alfa-Aesar, UK) were used for the preparation of buffer. The coloring agent ninhydrin, dinitrosalicylic acid (DNS) were from Sigma-Aldrich, USA. Trichloroacetic acid (TCA) and NaOH (Alfa-Aesar, UK) were used as reaction stopper. Acetic acid, EDTA and urea (BDH, England) were taken as chemical agents for determining the effect of enzymatic activity, on the other hand, NaCl, KCl, CaCl_2_, MgCl_2_, ZnCl_2_ and FeCl_2_ were used as metallic ion effect on enzyme activity.

### 2.3 Enzyme activity assay

The barley seeds were sorted, rinsed with distilled water and sanitized with 1 % calcium hypochlorite solution for 10 min. Then the seeds were soaked for overnight. They were then dried at room temperature. After that, the seeds were made flour using an electric blender. The flour of seeds was used for the preparation of enzyme extract.

Since barley seeds contained cysteine proteases, they worked at an acidic pH range 4.5–6.6 [22]. Similarly, α-amylase from barley seeds had an optimal pH range of 6.5–7.0 [23]. For the protease samples, a pH of 5.5 was utilized, while for the amylase samples, a pH of 6.7 was employed to ensure the maximum enzyme activity throughout the experiment. About 2.0 g of seed flour was processed by crushing them into a paste using a homogenizer (mortar pestle). An amount of 12.0 mL respected buffer was added to it and mixed them appropriately. In order to maintain a constant temperature of 4 °C during experiment, an ice bowl was placed under the homogenizer. It also protected the enzyme denaturation during extraction. The suspension was subsequently subjected to filtration using multiple layers of cheesecloth (commercially known as trade ford) within a controlled cold room environment. The filtrate obtained from the initial process was subjected to further clarification through centrifugation. This was achieved by utilizing a refrigerated centrifuge operating at a speed of 10,000 revolutions per min (rpm) for a duration of 20 min and this condition provided the efficient separation of enzymes from cellular debris [24]. The entire process was carried out at a temperature of 4 °C. Then the supernatant was collected and discarded the residue. The crude enzyme extract obtained after centrifugation was directly used for subsequent enzymatic assays [25].

#### 2.3.1 Assay of α-amylase activity

Dinitrosalicylic acid (DNS) method was used to test the activity of a-amylase [25]. As a substrate, 1 % soluble starch (1.0 g in 100 mL of phosphate buffer, pH 6.7) was used. For each of the sample test tubes, 2.50 ml of phosphate buffer, 2.50 ml of starch substrate, 1 ml of 1 % NaCl solution, and 0.50 ml of enzyme extract were added. In a control test tube, 2.50 ml of phosphate buffer, 2.50 ml of starch substrate, 1 ml of 1 % NaCl solution, and 0.50 ml of enzyme extract were added, and the reaction was immediately stopped by adding 0.50 ml of 2N NaOH solution. A blank was made with the same reagents except enzyme extract. Blank, sample, and control test tubes were incubated in a water bath at 37 °C for 15 min. After the incubation period, 0.50 ml of 2 N NaOH solution was added to the blank and sample test tubes. Then 0.50 ml of DNS solution was added to all the test tubes and heated in the water bath for 5 min. The absorbances were measured at a wavelength of 520 nm. The determination of amylase activity was conducted by measuring the liberation of maltose. The quantification of maltose liberation was determined by applying the standard curve that was prepared using maltose. One unit of amylase activity was defined as the amount required to liberate 1 mg of maltose in 15 min at 37 °C.

#### 2.3.2 Assay of protease activity

Protease activity was determined by the modified method described by Asakura et al. [26]. As a substrate, 0.1 % haemoglobin was used (0.1 g in 100 mL of citrate buffer, pH 5.5). In a test tube, 0.50 ml of enzyme extract and 3.0 ml of 1 % haemoglobin were taken. The test tube was incubated in a water bath at 37 °C for 30 min. After the incubation period, the reaction was stopped with 3 ml of 5 % trichloroacetic acid, and then it was filtered with Whitman filter paper. After that, 0.50 ml of filtrate, 0.50 ml of distilled water, and 1 ml of ninhydrin solution were added to a sample test tube. 0.50 ml of citrate buffer, 0.50 ml of distilled water, and 1 ml of ninhydrin solution were added to another test tube, which was called a blank. Both the sample and the blank test tube were heated in a water bath for 20 min. Then the test tubes were removed from the heating, and the test tubes were cooled. 5 ml of diluent (n-propanol: H_2_O = 1:1) was added to all the test tubes. The absorbances were measured at a wavelength of 570 nm. The amount of leucine release was determined by utilizing a standard curve that was prepared using leucine. In this study, a unit of protease activity was precisely defined as the quantity of enzyme necessary to release 1 mg of leucine within a 30 min timeframe at a temperature of 37 °C.

### 2.4 Effect of chemical agents and metallic salts

In this study, amylase and protease enzymes extracting solutions from Barley were treated with CaCl_2_, urea, EDTA, acetic acid, and various metallic salts at different concentrations and the activities were investigated. The experiment involved adding these treatments to 5.0 ml of the enzyme extract solutions, followed by incubation for a duration of 10 min at a temperature of 20 °C. The mixtures were once again subjected to incubation alongside their corresponding substrate for a duration of 15 min at a temperature of 37 °C. Following this incubation period, the enzyme activities were assayed [27].

### 2.5 Statistical analysis

The importance of the changes in enzyme activity noted under different experimental settings was evaluated by statistical analysis. In this study, one-way ANOVA (analysis of variance) was performed to figure out the significance differences. As a post-hoc analysis, Duncan's Multiple Range Test (DMRT) was used to compare between treatment groups at a significance level of p < 0.05. On the other hand, Student's t-test was utilized for pairwise comparison between two groups at the same significance level. The results were shown as mean ± standard deviation, and each experiment was done three times. Statistical analysis was performed utilizing IBM SPSS software (version 27.0), and the Origin software (version 8.5) was employed to create graphs.

## 3 Results and discussion

### 3.1 The impact of CaCl_2_ on the enzymatic activities of α amylase and protease

The relative activity of the two enzymes showed a clear relationship with the CaCl_2_ concentration (Table 1). The activities of α-amylase and protease were set at 100.00 % at 0.0 M CaCl_2_. Nevertheless, enzyme activities were found to rise in a concentration-dependent manner upon the addition of CaCl_2_. The activity of α-amylase reached 105.36 ± 0.73 % and protease activity 106.86 ± 1.07 % at 0.001 M CaCl_2_. This pattern persisted even when the CaCl_2_ concentration was increased further. Notably, in the presence of a calcium concentration of 0.50 M, the maximum activities of α-amylase and protease were observed to be 137.26 ± 1.42 % and 135.65 ± 1.17 % respectively. Similar calcium-stimulating effects on enzyme activity in different biological systems have been described in earlier research [28]. These findings suggest a positive correlation between calcium concentration and enzyme activities, highlighting the potential regulatory role of calcium in enzymatic processes.

**Table 1:** Effects of calcium chloride on the activities of amylase and protease.

Concentration of CaCl_2_ (Molar)	Relative activities (%)	
	Alpha amylase	Protease
0	100	100
0.001	105.36 ± 0.73^a^	106.86 ± 1.07^a^
0.005	109.44 ± 0.70^b^	109.44 ± 1.11^b^
0.01	115.50 ± 0.88^c^	114.65 ± 0.82^c^
0.05	119.11 ± 0.53^d^	118.28 ± 0.96^d^
0.10	125.64 ± 0.54^e^	122.96 ± 1.01^e^
0.30	131.24 ± 0.48^f^	128.64 ± 0.85^f^
0.50	137.26 ± 1.42^g^	135.65 ± 1.17^g^
0.60	136.83 ± 1.23^g^	135.13 ± 0.98^g^
0.70	136.73 ± 1.09^g^	134.56 ± 0.82^g^
0.80	137.13 ± 1.08^g^	134.36 ± 0.87^g^
0.90	135.83 ± 1.66^g^	134.95 ± 1.08^g^
1.00	136.56 ± 1.38^g^	135.41 ± 1.42^g^

Values are expressed as mean ± standard deviation (n = 3). Different letters in the same column indicate significant (p < 0.05) differences.

### 3.2 Enzyme activity of α-amylase and protease upon exposure to varying concentrations of urea

**Fig. 1:**
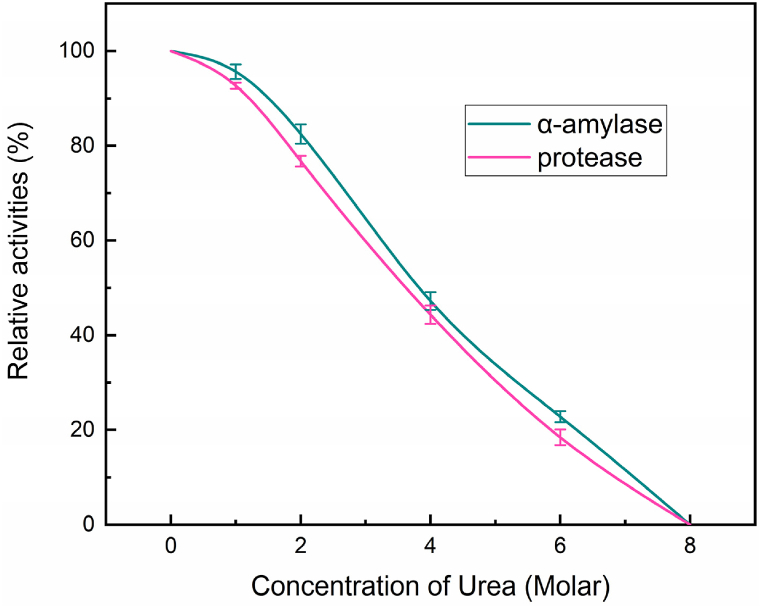
Effects of urea on the activities of α-amylase and protease.

With respect to different molar concentrations, the effect of urea on the activities of protease and α-amylase enzymes in barley was investigated, as illustrated in Fig. 1. The activity of both enzymes showed a strong correlation with the concentration of urea. The α-amylase and protease activities were set at 100.00 % at a foundational concentration of 0.00 M urea. On the other hand, as the concentration of urea increased, enzyme activities decreased noticeably. When exposed to a concentration of 1.00 M urea, the activity of α-amylase reduced to 95.64 ± 1.54 %, while the activity of protease declined to 92.68 ± 0.64 %. This pattern persisted as the concentration of urea was further increased. When exposed to a concentration of 8.00 M urea, both the α-amylase and protease activities were entirely eliminated, indicating a total absence of enzyme functioning. These results showed that urea inhibited α-amylase and protease enzymes. Urea is recognized for its ability to disturb the structure and function of proteins by causing denaturation through the breaking of hydrogen bonds and hydrophobic interactions [29]. Protein denaturation by urea explains the decrease in enzyme activity with increasing urea concentration.

### 3.3 Enzyme activity of α amylase and protease upon exposure to varying concentrations of EDTA

**Fig. 2:**
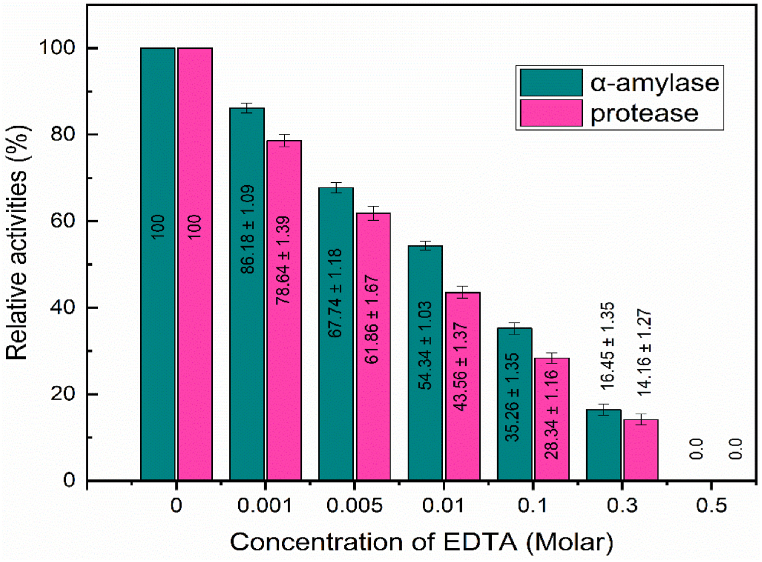
Effects of EDTA on the activities of α-amylase and protease.

EDTA affected the activities of α-amylase and protease enzymes in barley at different molar concentrations (Fig. 2). The activities of α-amylase and protease were 100.00 % at 0.00 M EDTA. When the amounts of EDTA increased, a notable decline in enzyme activity was detected. At 0.001 M EDTA, α-amylase activity dropped to 86.18 ± 1.09 % and protease activity to 78.64 ± 1.39 %. As the EDTA concentration was further raised, this negative tendency remained. Complete inhibition of α-amylase and protease activity was seen at 0.500 M EDTA. EDTA is a chelating chemical that is recognized for its capacity to attach to metal ions, therefore preventing the functioning of enzyme's activity [30].

### 3.4 Enzyme activity of α-amylase and protease upon exposure to varying concentrations of acetic acid

**Fig. 3:**
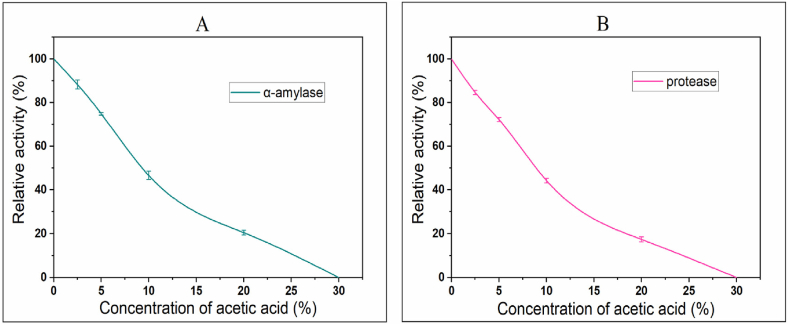
Effects of acetic acid on the activities of the enzymes (A) α-amylase and (B) protease.

The effects of acetic acid on the enzymatic activities of α-amylase and protease in barley were investigated at different concentrations (Fig. 3). The activities of both enzymes were noticeably influenced by the concentration of acetic acid. When the concentration was initially 0 %, the activities of α-amylase and protease were established at 100.00 %. Nevertheless, when the concentration of acetic acid increased, a significant decline in enzyme activity was noted. When exposed to a concentration of 2.50 % acetic acid, the activity of α-amylase reduced to 88.34 ± 1.98 %, while the activity of protease declined to 84.68 ± 0.99 %. This pattern persisted as the level of acetic acid concentration was further increased. At a concentration of 30.00 % acetic acid, the activities of both α-amylase and protease were completely eliminated. The results highlight the suppressive impact of acetic acid on the functions of α amylase and protease enzymes in barley. Acetic acid may bind to a site on the enzyme other than the active site, preventing it from forming the enzyme-substrate complex at its normal rate [31]. The protein denaturing effect of acetic acid explains the decrease in enzyme activity with increasing concentration.

### 3.5 The impact of metallic salts on the enzymatic activity of α amylase and protease

From Table 2, It was shown that MgCl_2_ increased α-amylase and protease activity depending on its concentration. The activity of α-amylase jumped to 105.82 ± 2.21 % at 0.001 M and 109.36 ± 1.26 % at 0.002 M. Magnesium ions may be enzyme cofactors or stabilizers. MgCl_2_ also improved protease activity which was 107.10 ± 1.28 % at 0.001 M and 110.25 ± 1.87 % at 0.002 M. It appears that magnesium ions stabilize protease enzymes. MnCl_2_ enhanced α-amylase and protease activities significantly. The activity of α-amylase exacerbated at both concentrations, peaking at 127.36 ± 1.84 % at 0.002 M. The manganese ion improved catalytic efficiency or stability. MnCl_2_ also increased protease activity in a concentration-dependent manner, with 0.002 M having the highest relative activity of 122.74 ± 1.33 %. In contrast, ZnCl_2_, CuCl_2_, and FeCl_2_ demonstrated inhibitory effects on α-amylase and protease activity at varied molar concentrations. The activity of α-amylase and protease were 86.28 ± 1.32 % and 90.62 ± 0.02 % respectively, at 0.001 M ZnCl_2_. Increasing the concentration to 0.002 M lowered α-amylase activity to 74.22 ± 1.72 % and protease activity to 86.48 ± 0.97 %. For CuCl_2_ at 0.001 M α-amylase and protease activity were 86.72 ± 0.86 % and 72.82 ± 1.92 %. The activity of α-amylase reduced dramatically to 77.18 ± 1.15 % at 0.002 M, while protease activity declined to 54.64 ± 0.71 %. Table 2 also displayed the impact of FeCl_2_. The activity α-amylase and protease were 74.26 ± 1.78 % and 66.38 ± 1.11 % respectively at 0.001 M FeCl_2_. α-amylase activity dropped to 60.56 ± 0.96 % and protease activity to 54.32 ± 0.98 % at 0.002 M [32]. It demonstrated the concentration-dependent effects of ZnCl_2_, CuCl_2_, and FeCl_2_ on α-amylase and protease enzymes, suggesting that these three ions strongly block the catalytic activities of both enzymes. It is worth mentioning that the activities of α-amylase or protease were not significantly affected by NaCl and KCl. This highlights the specificity of the observed effects with other metallic salts and eliminates the possibility of changes caused by differences in ionic strength.

**Table 2:** The impact of various metallic salts on the activity of α-amylase and protease.

Metallic salts	Concentration (Molar)	Relative activities (%)	
		Alpha amylase	Protease
None	–	100.00	100.00
MgCl_2_	0.001	105.82 ± 2.21^a^	107.10 ± 1.28^a^
	0.002	109.36 ± 1.26^b^	110.25 ± 1.87^b^
ZnCl_2_	0.001	86.28 ± 1.32^a^	90.62 ± 1.85^a^
	0.002	74.22 ± 1.72^b^	86.48 ± 0.97^b^
CuCl_2_	0.001	86.72 ± 0.86^a^	72.82 ± 1.92^a^
	0.002	77.18 ± 1.15^b^	54.64 ± 0.71^b^
MnCl_2_	0.001	118.64 ± 1.85^a^	114.26 ± 1.80^a^
	0.002	127.36 ± 1.84^b^	122.74 ± 1.33^b^
NaCl	0.001	100.00 ± 0.22^a^	99.46 ± 0.74^a^
	0.002	99.78 ± 0.13^a^	98.16 ± 0.83^a^
KCl	0.001	100.00 ± 0.00^a^	100.00 ± 0.00^a^
	0.002	100.00 ± 0.00^a^	100.00 ± 0.00^a^
FeCl_2_	0.001	74.26 ± 1.78^a^	66.38 ± 1.11^a^
	0.002	60.56 ± 0.96^b^	54.32 ± 0.98^b^

Values are expressed as mean ± standard deviation (n = 3). Different letters in the different concentration of each salt indicate significant (p < 0.05) differences.

## 4 Conclusion

The study examined the complicated effects of chemical agents and metallic ions on the activities of α-amylase and protease enzymes in barley (*Hordeum vulgare* L.). The results elucidate the different effects of these treatments and provide scientific and industrial insights. According to the study, calcium in its metallic salt form boosts enzyme activity. Calcium may be important in controlling these actions. Urea and EDTA significantly decreased α-amylase and protease activity. These enzymes were fully inhibited at greater concentrations. Acetic acid treatment gradually decreased enzyme activity, leaving just a fraction at 20 % concentration. After significant research, metallic salts affected α-amylase and protease activities in interesting ways. The effects were augmented by MgCl_2_, indicating that it might serve as a cofactor or stabilizer. The investigation revealed that MnCl_2_ had a substantial impact on enhancing enzyme activity. Nevertheless, ZnCl_2_, CuCl_2_, and FeCl_2_ hindered the activities of the enzyme at varying concentrations. Whereas the presence of NaCl and KCl did not have a substantial impact on enzyme activity, emphasizing their distinct effects. These findings affect agriculture, biotechnology, and environmental sciences. This information can help develop plans to mitigate the detrimental impacts of metal stress on crop yield and food safety in agriculture. This research sheds light on the complicated interactions between chemical agents and enzymes, which can improve enzymatic processes in several sectors.

## CRediT authorship contribution statement

**Amin Hossain:** Writing – original draft, Visualization, Software, Methodology, Investigation, Formal analysis, Data curation. **Umma Fatema Shahjadee:** Writing – review & editing, Supervision, Methodology, Investigation, Data curation. **Abu Tareq Mohammad Abdullah:** Writing – review & editing, Supervision. **Mohammad Nazrul Islam Bhuiyan:** Writing – review & editing, Supervision. **Anjum Zerin Rupa:** Writing – review & editing, Methodology, Investigation.

## Ethical statement

Approval by an ethics committee was not needed for this study because this study did not include any humans or animals.

## Data availability statement

Data will be made available on request. For requesting data, please write to the corresponding author.

## Declaration of competing interest

The authors declare that they have no known competing financial interests or personal relationships that could have appeared to influence the work reported in this paper.
